# Alterations of Diffusion Kurtosis and Neurite Density Measures in Deep Grey Matter and White Matter in Parkinson’s Disease

**DOI:** 10.1371/journal.pone.0157755

**Published:** 2016-06-30

**Authors:** Yulia Surova, Björn Lampinen, Markus Nilsson, Jimmy Lätt, Sara Hall, Håkan Widner, Swedish BioFINDER study, Danielle van Westen, Oskar Hansson

**Affiliations:** 1 Department of Clinical Sciences, Lund, Lund University, Lund, Sweden; 2 Department of Neurology, Skåne University Hospital, Lund, Sweden; 3 Department of Medical Radiation Physics, Lund University, Lund, Sweden; 4 Lund University Bioimaging Center, Lund University, Lund, Sweden; 5 Center for Medical Imaging and Physiology, Skåne University Hospital, Lund, Sweden; 6 Department of Clinical Sciences, Malmö, Lund University, Malmö, Sweden; 7 Memory Clinic, Skåne University Hospital, Malmö, Sweden; University Medical Center Utrecht, NETHERLANDS

## Abstract

In Parkinson’s disease (PD), pathological microstructural changes occur and such changes might be detected using diffusion magnetic resonance imaging (dMRI). However, it is unclear whether dMRI improves PD diagnosis or helps differentiating between phenotypes, such as postural instability gait difficulty (PIGD) and tremor dominant (TD) PD. We included 105 patients with PD and 44 healthy controls (HC), all of whom underwent dMRI as part of the prospective Swedish BioFINDER study. Diffusion kurtosis imaging (DKI) and neurite density imaging (NDI) analyses were performed using regions of interest in the basal ganglia, the thalamus, the pons and the midbrain as well as tractography of selected white matter tracts. In the putamen, the PD group showed increased mean diffusivity (MD) (*p* = .003), decreased fractional anisotropy (FA) (*p* = .001) and decreased mean kurtosis (MK), compared to HC (*p* = .024). High MD and a low MK in the putamen were associated with more severe motor and cognitive symptomatology (*p* < .05). Also, patients with PIGD exhibited increased MD in the putamen compared to the TD patients (*p* = .009). In the thalamus, MD was increased (*p* = .001) and FA was decreased (*p* = .032) in PD compared to HC. Increased MD and decreased FA correlated negatively with motor speed and balance (*p* < .05). In the superior longitudinal fasciculus (SLF), MD (*p* = .019) and *f*_iso_ were increased in PD compared to HC (*p* = .03). These changes correlated negatively with motor speed (*p* < .002) and balance (*p* < .037). However, most of the observed changes in PD were also present in cases with either multiple system atrophy (n = 11) or progressive supranuclear palsy (n = 10). In conclusion, PD patients exhibit microstructural changes in the putamen, the thalamus, and the SLF, which are associated with worse disease severity. However, the dMRI changes are not sufficiently specific to improve the diagnostic work-up of PD. Longitudinal studies should evaluate whether dMRI measures can be used to track disease progression.

## Introduction

Parkinson’s disease (PD) is the second most common neurodegenerative disorder, with a prevalence of approximately 1% in the population older than 60 years. It is classically characterized by resting tremor, slowness of initial movement, rigidity, and general postural instability [1]. Microstructural changes include neuronal loss in the substantia nigra [2]. The loss of cells results in profound dopamine depletion in the motor region of the striatum [3], with nigral projections to the putamen being most affected [4]. Neuropathology reports in PD have shown that the thalamus and the putamen are also affected [4, 5]. Post-mortem diagnosis of PD requires evidence of cell loss in the substantia nigra, aggregated α-synuclein deposits accumulated in neurites (Lewy neurites) and in neuronal somata (Lewy bodies). The clinical signs and symptoms of PD vary considerably, and are assumingly caused by differences in the degeneration pattern of the nigrostriatal dopaminergic system and other subcortical neuronal systems [6–8]. A number of proposals divide patients with PD into the tremor dominant (TD) and postural instability gait difficulty (PIGD) subtypes [6, 7]. As of yet, no study has assessed if there are differences in measures from diffusion MRI (dMRI) between PIGD and TD.

Conventional magnetic resonance imaging (MRI) has been unsuccessful in detecting pathophysiologic changes in PD. However, several changes have been found in measures from dMRI techniques. Using diffusion tensor imaging (DTI), spatially resolved micro-structural brain damage has been identified in PD [9]. Overall, DTI measures tend to show consistent results of changes in brain regions involved in the basal ganglia circuit, including an elevation of mean diffusivity (MD) and a reduction of fractional anisotropy (FA) [9, 10]. However, a recent meta-analysis of FA changes in the substantia nigra has questioned the stability and validity of this measure as a PD biomarker [10]. Several studies have reported increased MD and reduced FA in the caudate nucleus, the putamen, the globus pallidus and the thalamus in PD [11–16]. Other reports have shown normal dMRI in the striatum of PD [17, 18]. Together these somewhat divergent results call for large prospective studies to investigate dMRI changes in PD. On the other hand, the patterns of change seem to be more robust in atypical parkinsonian disorders like progressive supranuclear palsy (PSP) or multiple system atrophy (MSA), with changes in dMRI measures reported in the basal ganglia regions such as the putamen and the thalamus [19–25], but also in the caudate nucleus and the globus pallidus [14, 26, 27]. Interestingly, DTI measures in the basal ganglia are promising for differentiating atypical parkinsonian disorders from PD [10].

Changes in FA and MD have also been observed in several brain white matter (WM) tracts in PD. Compared to healthy controls (HC), patients with PD exhibit increased MD and reduced FA in the superior longitudinal fasciculus (SLF), the genu of the corpus callosum (CC), and in the cingulum (CG) [28]. Interestingly, Zheng et al. [29] showed that executive function, linguistic performance, attention, and memory were positively correlated with FA and negatively correlated with MD in relevant WM tracts, consistent with the expectation that FA decreases and MD increases with increased levels of neurodegeneration and neurocognitive dysfunction.

Changes in diffusion kurtosis, which quantifies non-Gaussian water diffusion [30], have not yet been well studied in PD. Wang et al. [24] demonstrated elevated mean kurtosis (MK) in the putamen and in the substantia nigra in PD. Reduced FA and MK have been found in PD patients in WM structures such as the cingulate fiber tracts [28], the anterior part of the inferior fronto-occipital fasciculus (IFOF), the anterior SLF, the anterior and superior corona radiata, parts of the genu and body of the CC, and part of the parietal WM (part of the posterior SLF) [28].

In this explorative study, we aimed to extend the previously reported findings of dMRI changes in grey matter (GM) and brain WM tracts in PD. To this end, multi-shell dMRI data were obtained from a large sample of PD patients, as well as from healthy controls. The data were analyzed using diffusion kurtosis imaging (DKI) to obtain MD, FA and MK, and neurite density imaging (NDI) [31] to obtain the neurite density index (*f*_ic_) and the partial fraction of free water (*f*_iso_). Values of these parameters were obtained in GM structures by drawing regions of interest (ROI) in the basal ganglia, the thalamus, the midbrain and the pons. In WM, parameter values were obtained in tracts defined using tractography and included the cingulum (CG), the CC, the fornix, the SLF, the inferior longitudinal fasciculus (ILF), the IFOF, the uncinate fasciculus (UF), and the corticospinal tract (CST). First, we investigated whether diffusion changes in subcortical GM structures and WM tracts could be found in PD compared to HC, and found changes in putamen, thalamus, and SLF. Second, we studied whether these changes were different between the PIGD and TD subtypes, as well as between PD and patients with PSP or MSA. Third, we tested whether the diffusion changes in PD were associated with motor or cognitive deficits.

## Materials and Methods

### Ethics Statement

This study was approved by the Ethics Committee at Lund University and performed in accordance with the Helsinki Declaration. All participants gave written informed consent prior to participation.

### Participants

In this case-control study participants were recruited from the Neurology Clinic at Skåne University Hospital, Sweden, between 2008 and 2015 as part of the prospective and longitudinal Swedish BioFINDER study (http://www.biofinder.se) [32]. For the present work, 105 subjects were included with a clinical diagnosis of probable PD. The diagnosis was made by neurologists trained in movement disorder diagnostics according to the National Institute of Neurological Disorders and Stroke (NINDS) criteria of PD [33]. Neurologically HC were recruited (n = 44), who did not have any objective cognitive or parkinsonian symptoms. In addition, we included patients with a clinical diagnosis of probable PSP (n = 10) and MSA (n = 11) who fulfilled the NINDS criteria of either PSP or MSA [34, 35]. Motor function, disease stage and disability was evaluated using e.g. Unified Parkinson’s disease rating scale motor part (UPDRS-III) [36], Hoehn and Yahr staging scale (H&Y), the Schwab and England activities of daily living scale (S&E) [37], and the timed up and go test [38, 39]. The tandem gait test [40] was done to assess disturbances in balance and gait. Cognitive assessments were conducted by trained physicians using a standardized battery, including Mini Mental State Examinations (MMSE), The Quick Test of Cognitive Speed (AQT) test [41], and the memory subtests of the Alzheimer's Disease Assessment Scale (ADAS-Cog, which consist of a 10 word delayed recall). To ensure standardization, assessments were conducted during patients “on” medication state, or fully responding to their PD medications (in the “on” state). At the time of testing, none of the patients exhibited any dyskinesia, dystonia, or other signs of involuntary movement.

#### Classification into postural instability gait disorder and tremor dominant subtypes of patients with Parkinson’s disease

The UPDRS-III motor score was used to compute a mean tremor score of the following 9 tremor items: right and left arm tremor as determined by history, during patients “on” and “off” medication state. Tremor at rest of either lips, face or chin tremor, tremor in all 4 limbs, and action or postural tremor on both arms were determined by the investigator during examination. A mean score of 5 PIGD items were computed: falling, freezing, and walking difficulty by history, during patients “on” and “off” medication state. Gait and postural instability were determined during patients’ examination). When the ratio of the mean tremor score divided by the PIGD score was greater than or equal to 1.5, study participants were assigned to the TD subgroup [6]. When this ratio was equal to or less than 1.0, study participants were assigned to the PIGD subgroup [6]. Patients with mixture phenotype were ignored when comparing PIGD to TD.

### MRI data acquisition and post-processing

Imaging was performed on a 3 T Siemens Skyra MR scanner equipped with a 20 channel head coil. The diffusion MRI protocol comprised 99 DWI volumes, where the choice of *b*-values and encoding directions was inspired by Poot et al. [42]. In total, three volumes were acquired with *b* = 0 s/mm^2^, while the remaining 96 volumes were acquired using *b*-values of 250, 500, 1000, and 2750 s/mm^2^, distributed over 6, 6, 20, and 64 directions, respectively. A single-shot spin-echo with EPI read-out was used, with the following settings: TR = 8100 ms, TR = 103 ms, voxel size = 2.3×2.3×2.3 mm^3^, FOV = 294×294×120 mm^3^, iPAT = 2, and partial Fourier factor = 6/8. The imaging volume comprised 52 contiguous axial slices adjusted to include the whole cerebrum. Total acquisition time was approximately 14 minutes. Motion and eddy current distortions were corrected using an extrapolation-based method for improved high *b*-value performance [11]. In this procedure, the diffusion-weighted images were modulated with the Jacobian determinant of the transformation matrix [43]. In order to mitigate the potential effects of Gibbs ringing artifacts, image volumes were smoothed using an isotropic 3D Gaussian kernel with a full-width at half maximum of 2.3 mm [44–46]. Smoothing with a kernel of this size has little effect on sensitivity and specificity [47], and is thus not expected to significantly influence the parameter precision. DKI analysis was performed to obtain maps of FA, MK, and MD, using in-house developed software which fitted the diffusion and kurtosis tensors by nonlinear optimization as in Lätt et al [48]. The fitting only allowed positive values of the diffusion tensor eigenvalues. In a small number of voxels where the kurtosis was below zero, the fitting was repeated after additional smoothing was performed. NDI analysis [31], a simplification of the NODDI analysis [49], was performed to obtain maps of the neurite density index (*f*_ic_) and the partial fraction of free water (*f*_iso_). NDI utilizes a concept from solid-state NMR called powder averaging [50], where the MR signal is averaged across all rotations of a sample. In dMRI, we average across encoding directions for each b-value, which induces complete orientation dispersion in each voxel. This concept has previously been applied in diffusion MRI to analyze microscopic diffusion anisotropy [51, 52]. Here, we use it to simplify the NODDI model in order to speed up the analysis by predicting the MR signal in terms of only three model parameters (*S*_0_, *f*_ic_, and *f*_iso_), according to

$$S=S_{0}\left(f_{\text{iso}}\text{exp}\left(-bD_{\text{iso}}\right)+\left(1-f_{\text{iso}}\right)\left(f_{\text{ic}}A_{\text{ic}}+\left(1-f_{\text{ic}}\right)A_{\text{ec}}\right)\right)$$ (1)

where the attenuation A of the intra-neurite and extracellular components were given by

$$A_{x}=\text{exp}\left(-b{\text{MD}}_{x}\right)h\left(\alpha_{x}\right)$$ (2)

and

$$h\left(\alpha \right)=\sqrt{\frac{\pi }{4\alpha }}\text{exp}\left(\alpha /3\right)\text{erf}\left(\sqrt{\alpha }\right)$$ (3)

Here, erf is the error function and α_*x*_ = *b* (AD_x_–RD_x_) where AD_x_ and RD_x_ are the axial and radial diffusivities, respectively, from which the mean diffusivity is calculated according to MD_x_ = (AD_x_ + 2 RD_x_)/3. [53]. Just as for the NODDI model, we assumed AD_ic_ = AD_ec_ = 1.7 μm^2^/ms, RD_ic_ = 0, and RD_ec_ = (1–*f*_ic_) AD_ec_. Just as for NODDI, NDI is built on the assumption that the diffusivities are identical inside and outside the axons, which is not necessarily true and may thus bias the estimated parameters [54, 55]. Accordingly, the values of the obtained parameters should be interpreted as phenomenological fit parameters rather than in terms of absolute quantification of specific features of the tissue microstructure.

All calculations were performed using in-house developed software, implemented in Matlab (The Mathworks, Natick, MA, USA).

All relevant data are within the paper. The raw imaging data are available for researchers who meet the criteria for access to confidential data and are granted accessed by the local data access committee.

### Region of interest analysis of diffusion parameters

#### ROI based analysis of grey matter

**Fig 1:**
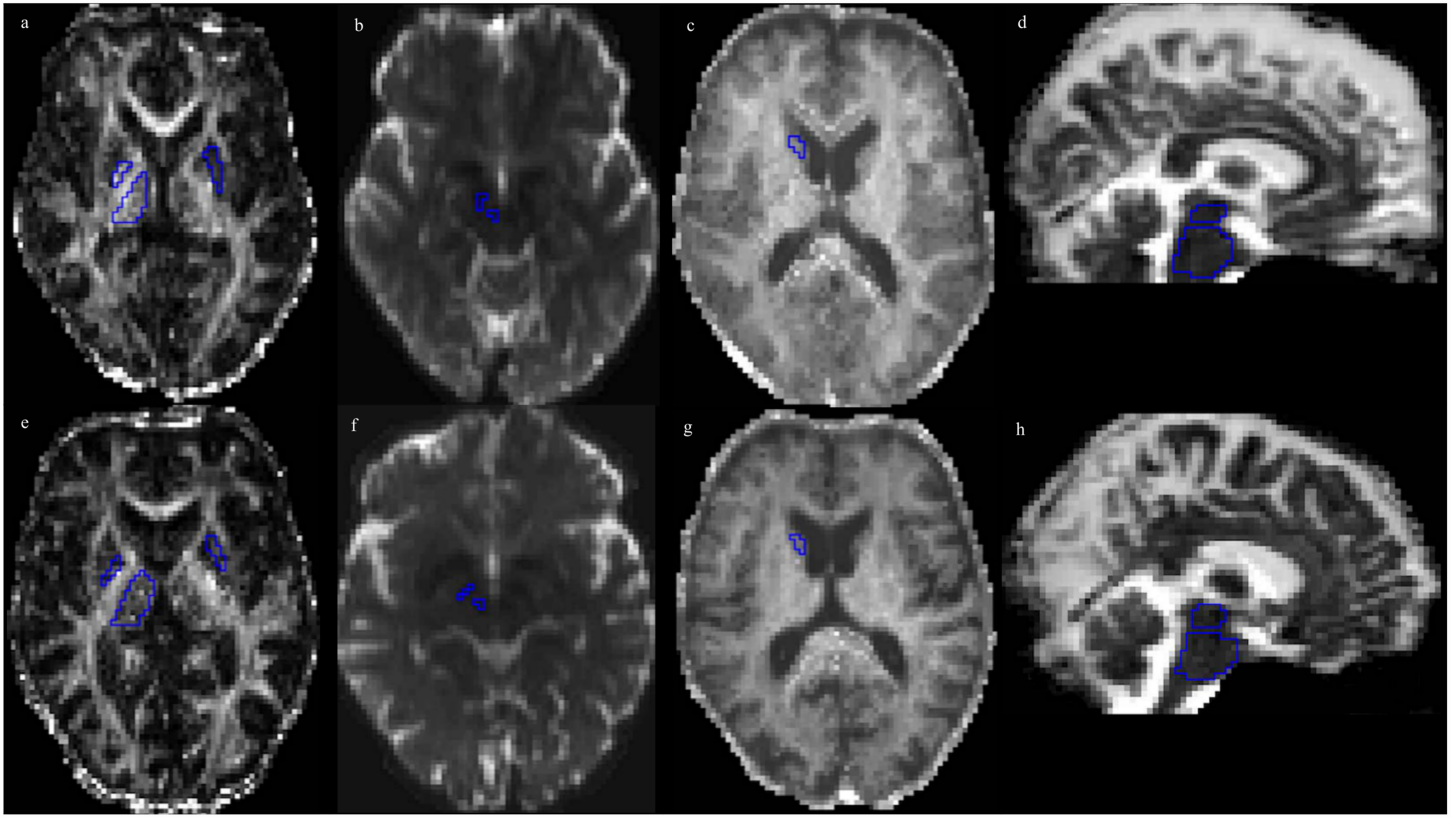
Parameter maps for both PD patients and healthy controls with key ROIs examined. ROIs for measurement in the anatomical areas reported in Table 2, overlaid on the FA, *b*_0_, MK and MD maps from a 66-year-old healthy male participant (panels a-e), and a 66-year-old male PD patient (panels f-j). In panels (a) and (f), ROIs are placed in the pallidum and the thalamus (right), and the putamen (left) on the FA-map; in panels (b) and (g) in the substantia nigra and red nucleus (right) on the *b*_0_-map; in panels (c) and (h) in the caudate head (right) on the MK map; and in panels (d) and (i) in the pons and midbrain on the MD map.

All ROIs were outlined manually (Fig 1) in the subject’s native space using MK and directionally color-encoded FA (DEC) maps, with the exception of the red nucleus and the substantia nigra, where the non-diffusion weighted *b*_0_ map was used. ROI size was adjusted in order to maximize coverage of each structure in each subject, while minimizing partial-volume effects from neighboring areas. Contamination from cerebrospinal fluid (CSF), which has isotropic diffusion with a high MD, was avoided by excluding voxels adjacent to the third and lateral ventricles. Slice selection for ROI delineation was performed with respect to standard neuroanatomical criteria [56]. The head of the caudate nucleus was delineated in one slice at the level where it was most conspicuous. The thalamus was delineated in 5–8 consecutive slices at the level of the internal capsule and adjacent to the interthalamic adhesion. The globus pallidus and the putamen were delineated in one slice, and were adjusted to the extreme capsule. The red nucleus was identified as a circular area of signal hypointensity in the midbrain on *b*_0_ maps and delineated in one axial slice. The substantia nigra was identified as an area of signal hypointensity in the midbrain on *b*_0_ maps and delineated in one axial slice. The midbrain and the pons were delineated in 8 consecutive sagittal slices, with the boundaries identified using the manual approach proposed by Oba et al. [57]. For each structure, the average MK, MD, FA value and NDI parameters from the right and left hemisphere was calculated. All ROIs were redrawn 8 weeks later on the same images, by the same investigator (intra-rater variability > 0.9 for all ROIs), and the average values from paired ROIs were used for analysis. The image-presentation order was randomized, and the investigator was blinded to the order. In the substantia nigra, red nucleus and globus pallidus, and midbrain, the signal-to-noise ratio (SNR) was approximately 50% of that in the thalamus, and 25–35% of that in WM (data not shown). The low SNR in these structures was suspected of causing artificial changes in FA, MD and MK (S1 File, S1 Fig), wherefore the structures were excluded from the analysis, besides the substantia nigra.

#### Tractography of brain white matter tracts

Tractography was performed using deterministic tracking based on constrained spherical deconvolution (CSD) [58], generating the left and right anterior half of the dorsal CG, the posterior half of the CG, the hippocampal CG, the SLF, the ILF, the IFOF, the UF, the CST and the CC. The fornix was generated using probabilistic tractography. The ROIs used to define the seed region for each tract, and to segment the tract based on logical operations, were defined in MNI152 standard-space [59] and warped back to native space utilizing the warp-fields generated by FLIRT and FNIRT [60].

All tracts were generated using one seed-ROI covering the full extent of the expected tract, and then further defined using logical AND- and NOT-ROIs. The subdivisions of the CG were defined according to Jones et al. [61], using two NOT-ROIs, one at its most anterior border and one at the rostral-caudal midpoint of the dorsal CG. The posterior CG was defined using two NOT-ROIs, one at the rostral-caudal midpoint of the dorsal CG and one at the dorsal border of the splenium. The hippocampal CG was defined using two NOT-ROIs, one at the dorsal border of the splenium of the CC and one at the level of the mesencephalon. The fornix was defined using two AND-ROIs and one NOT-ROI. The first AND-ROI was located at the level of the corpus fornicis, inferior to the anterior pillars, and the second was located at the level of the crus fornicis, inferior to the splenium of the CC. The NOT-ROI was located anterior to the anterior pillars, inferior to the crus fornicis and through the CC. The CC was defined using NOT-ROIs located just above its anterior, posterior, superior and inferior borders. Furthermore, AND-ROIs were used to include its 2 cm wide central portion. The SLF was defined according to Wakana et al. [62], using one AND-ROI in the frontal lobe at the level of the rostral-caudal midpoint of the CC, and one AND-ROI located just anterior to the posterior border of the Sylvian fissure. The ILF was defined using one AND-ROI anteriorly in the temporal lobe and one AND-ROI in the occipital lobe 2 cm posterior to the sagittal stratum. The IFOF was defined using one AND-ROI in the frontal lobe located inferior to the anterior-inferior border of the CC and one AND-ROI in the occipital lobe at the level of the sagittal stratum. The UF was defined using two AND-ROIs, one located in the frontal lobe 1 cm anterior to the CC, and one located in the temporal lobe at the level of central pons. In addition, a NOT-ROI was placed in the midsagittal plane, as well as posterior to the UF In addition, for the SLF, the ILF, the IFOF and the UF, a NOT-ROI was placed in the midsagittal plane. The CST was defined using one AND-ROI located in the thalamus and a second AND-ROI located in the motor cortex at the level of the hand area.

The average parameter estimates for each WM tract were used in the subsequent analysis.

### Statistical analysis

Statistical analysis was performed with SPSS Statistics 20 for Windows (IBM Corporation, Somers, NY, USA). Using the Mann-Whitney *U* test, comparisons of demography, clinical categorical variables and dMRI parameters were performed for: PD vs. HC, PIGD vs. TD, PD vs. PSP as well as PD vs. MSA. Correlations between diffusion parameters and clinical scores were tested for using Spearman’s rho (R_s_). Proportions were compared using Pearson’s chi-squared test. For the dMRI parameter comparisons that came out significant using non-parametric testing, a one-way analysis of covariance (ANCOVA) was also conducted in order to control for the effects of gender and age. MD in the thalamus, MK and FA in the putamen, MD and *f*_iso_ in the SLF, and *f*_ic_ in CST were all non-normally distributed, hence logarithmic transformation was performed before analysis with ANCOVA. The significance level was set to 0.05. In addition, the effect sizes were calculated for significant changes in dMRI parameters.

Univariate binary logistic regression analysis was performed to study the ability of DKI measures to distinguish PD from HC. Based on the ANCOVA, significantly different dMRI parameters were assigned to binary logistic regression analysis. The model where PD was compared to HC was adjusted for age and sex. Adjusting for sex was performed since the patient group had a larger proportion of men. To assess the usefulness of DKI as a diagnostic tool for individual cases, the sensitivity, specificity and the optimal cutoff level of the DKI values were calculated for selected regions with receiver operator characteristic curve analysis (ROC). Multiple comparison correction was not applied in this explorative study.

## Results

### Demographics

Demographics for the HC and PD groups are given in Table 1. The PD and HC groups did not differ significantly by age, but there was a significant difference in gender distribution (Pearson’s c^2^ = 5.4, *p* < .05), with a higher proportion of men in the PD group. The PIGD group performed worse on tests reflecting gait and balance (timed up and go and tandem gait tests) and a cognitive function (AQT) compared to the TD group.

**Table 1:** Demographic data and biomarker levels for the diagnostic groups.

	HC (n = 44)	PD (n = 105)	*p*-value (PD vs. HC)	PIGD (n = 47)	TD (n = 50)	*p*-value (PIGD vs.TD)
Gender female: male	25: 19	61: 44	0.02 ^a^	20:27	15:35	0.07 ^a^
Age (years)	66 ± 8	66 ± 11	0.475^b^	66 ± 11	66 ±11	0.767 ^b^
Disease duration (years)	ND	5 ± 4		5 ± 5	5 ± 3	0.266 ^b^
Hoehn and Yahr stage	ND	2 ± 1		2 ± 1	2 ± 1	0.001^b^
Schwab and England	100	90 ± 9	<0.001 ^b^	87 ± 11	93 ± 6	0.001^b^
UPDRS-III motor score	2 ± 2	13 ± 10	<0.001 ^b^	13 ± 11	13 ± 8	0.734 ^b^
Tandem gait test “on”	0.21 ± 0.5	0.66 ± 1	0.006 ^b^	0.96 ± 1.2	0.34 ± 0.6	0.005 ^b^
Timed up and go test “on” (seconds)	8 ± 1	10 ± 2	<0.00 ^b^	10 ± 3	9 ± 2	0.031 ^b^
MMSE score	28 ± 2	28 ± 2	0.887 ^b^	28 ± 2	28 ± 1	0.731 ^b^
Memory delayed recall (ADAS-Cog)	2 ± 2	4 ± 7	0.031 ^b^	3 ± 3	3 ± 2	0.901 ^b^
AQT	61 ± 14	70 ± 18	0.011 ^b^	73 ± 22	67 ± 15	0.024 ^b^

Values are given as mean ±SD. UPDRS-III, Unified Parkinson’s disease rating scale motor part; MMSE, Mini Mental State Examination test; ADAS-Cog, Alzheimer's Disease Assessment Scale; AQT, the Quick Test of Cognitive Speed (AQT) test; HC, healphy controls; PD, Parkinson’s disease; PIGD, postural instability gait difficulty; TD, tremor dominant; SD, standard deviation; ND, not done.

^a^ Pearson’s chi-square

^b^ Mann-Whitney U test.

### Differences in dMRI parameters in PD compared to HC

Table 2 shows the comparisons in GM structures between PD and HC. In the putamen, MD was increased with 8% and FA was decreased with 9% in PD compared to HC (Mann-Whitney *U* test, *p* < .003; ANCOVA, *p* < .005). The PD group also exhibited a 6% decrease of the MK in the putamen compared to HC (Mann-Whitney *U* test, p < .05; ANCOVA, *p <* .001). In the thalamus, MD was increased with 4%, and FA was decreased with 3%, in PD compared to HC (Mann-Whitney *U* test, *p* < .05; ANCOVA, *p* < .001, *p* < .05, respectively). There were no significant age- and gender-adjusted differences in dMRI parameters in the caudate head or the pons between the two groups. There were no significant differences (*p <* .05, ANCOVA) in NDI parameters in the caudate head, thalamus, putamen, and pons found in PD. In the caudate head, the *f*_*iso*_ was decreased with 2% and in the thalamus, *f*_iso_ was increased with 2% in PD (*p* < .05, Mann-Whitney U test, *p* > .05, ANCOVA).

**Table 2:** Diffusion magnetic resonance measures in patients with Parkinson’s disease (PD) and healthy controls (HC). The PD group was also divided into those with postural instability and gait difficulty (PIGD) and tremor-dominant (TD) phenotypes.

Structure	Parameter	HC (n = 44)	PD (n = 105)	*p*-value ^a^ (PD vs. HC)	PIGD (n = 47)	TD (n = 50)	*p*-value ^a^ (PIGD vs.TD)
Substantia nigra	FA	0.64 ± 0.10	0.62 ± 0.11	0.571	0.62 ± 0.1	0.62 ± 0.11	0.971
Caudate head	FA	0.23 ± 0.04	0.22 ± 0.04	0.390	0.22 ± 0.03	0.23 ± 0.04	0.170
Putamen	FA	0.23 ± 0.04	0.20 ± 0.03	**0.001** ^b^	0.20 ± 0.33	0.21 ± 0.03	0.170
Thalamus	FA	0.37 ± 0.02	0.36 ± 0.02	**0.032** ^b^	0.36 ± 0.02	0.36 ± 0.02	0.683
Pons	FA	0.52 ± 0.02	0.51 ± 0.02	0.524	0.51 ± 0.03	0.51 ± 0.02	0.829
Substantia nigra	MD	0.45 ± 0.12	0.48 ± 0.12	0.251	0.49 ± 0.1	0.48 ± 0.12	0.885
Caudate head	MD	0.80 ± 0.12	0.83 ± 0.14	0.138	0.84 ± 0.15	0.82 ± 0.14	0.269
Putamen	MD	0.78 ± 0.10	0.80 ± 0.14	**0.003** ^b^	0.88 ± 0.17	0.81 ± 0.10	**0.009** ^c^
Thalamus	MD	0.77 ± 0.05	0.80 ± 0.05	**<0.001** ^b^	0.82 ± 0.05	0.80 ± 0.05	0.052
Pons	MD	0.70 ± 0.04	0.71 ± 0.05	0.165	0.71 ± 0.05	0.70 ± 0.05	0.549
Substantia nigra	MK	ND	ND		ND	ND	
Caudate head	MK	0.92 ± 0.11	0.95 ± 0.11	0.253	0.94 ± 0.10	0.94 ± 0.11	0.776
Putamen	MK	1.24 ± 0.19	1.16 ± 0.15	**0.024** ^b^	1.12 ± 0.13	1.18 ± 0.17	0.101
Thalamus	MK	1.19 ± 0.09	1.16 ± 0.10	0.066	1.15 ± 0.10	1.17 ± 0.10	0.569
Pons	MK	1.52 ± 0.11	1.55 ± 0.15	0.238	1.53 ± 0.15	1.57 ± 0.16	0.356
Substantia nigra	NDI *f*_ic_	ND	ND		ND	ND	
Caudate head	NDI *f*_ic_	0.66 ± 0.08	0.62 ± 0.15	0.168	0.60 ± 0.15	0.63 ± 0.16	0.330
Putamen	NDI *f*_ic_	0.77 ± 0.13	0.74 ± 0.12	0.119	0.73 ± 0.11	0.74 ± 0.12	0.762
Thalamus	NDI *f*_ic_	0.72 ± 0.07	0.72 ± 0.07	0.874	0.72 ± 0.07	0.73 ± 0.07	0.423
Pons	NDI *f*_ic_	0.94 ± 0.05	0.95 ± 0.04	0.123	0.95 ± 0.04	0.95 ± 0.04	0.979
Substantia nigra	NDI *f*_iso_	ND	ND		ND	ND	
Caudate head	NDI *f*_iso_	± 0.08	0.10 ± 0.08	0.038	0.01 ± 0.09	0.01 ± 0.08	0.539
Putamen	NDI *f*_iso_	0.11 ± 0.08	0.12 ± 0.09	0.987	0.13 ± 0.11	0.11 ± 0.08	0.569
Thalamus	NDI *f*_iso_	0.09 ± 0.04	0.11 ± 0.03	0.024	0.11 ± 0.04	0.10 ± 0.03	0.784
Pons	NDI *f*_iso_	0.10 ± 0.03	0.11 ± 0.03	0.122	0.11 ± 0.03	0.11 ± 0.03	0.445

MD [10^-9 m^2/s]. Values are given as mean ± SD. FA, fractional anisotropy; MD, mean diffusivity; MK, mean kurtosis; NDI *f*_iso_ and *f*_ic_, neurite density imaging measures; ND, not done.

^a^ *p*, Mann-Whitney U test.

^b^ *p* < .05, ANCOVA (age/gender adjusted).

^c^ *p* < .05, ANCOVA (age adjusted).

P-values given in bold were significant when both using Mann-Whitney U test and ANCOVA.

The comparisons in WM tracts between PD and HC are presented in Tables 3 and 4. In SLF, the MD was increased with 4% and *f*_iso_ was increased with 9% (Mann-Whitney *U* test and ANCOVA, *p* < .05) in PD. In CST, *f*_ic_ was significantly decreased with 3% (Mann Whitney *U* test and ANCOVA, *p <* .05 both) (Table 4).

**Table 3:** Diffusion kurtosis imaging measures in white matter structures in patients with Parkinson’s disease (PD) and healthy controls (HC).

Region	Parameter	HC (n = 44)	PD (n = 105)	*p*-value ^a^(PD vs. HC)
Cingulum anterior	FA	0.35 ± 0.03	0.34 ± 0.05	0.927
Cingulum posterior	FA	0.49 ± 0.03	0.49 ± 0.03	0.987
Cingulum hippocampus	FA	0.38 ± 0.03	0.40 ± 0.04	0.041
Fornix	FA	0.30 ± 0.04	0.30 ± 0.04	0.753
Corpus callosum	FA	0.55 ± 0.03	0.56 ± 0.04	0.280
SLF	FA	0.46 ± 0.03	0.46 ± 0.03	0.731
ILF	FA	0.45 ± 0.04	0.44 ± 0.05	0.070
IFOF	FA	0.51 ± 0.03	0.50 ± 0.08	0.148
Uncinate Fasciculus	FA	0.39 ± 0.03	0.39 ± 0.03	0.224
CST	FA	0.40 ± 0.12	0.38 ± 0.13	0.141
Cingulum anterior	MD	0.90 ± 0.05	0.89 ± 0.10	0.874
Cingulum posterior	MD	0.80 ± 0.04	0.81 ± 0.05	0.208
Cingulum hippocampus	MD	0.87 ± 0.07	0.88 ± 0.07	0.728
Fornix	MD	1.75 ± 0.24	1.73 ± 0.25	0.744
Corpus callosum	MD	1.26 ± 0.11	1.26 ± 0.12	0.698
SLF	MD	0.81 ± 0.05	0.84 ± 0.05	**0.019** ^b^
ILF	MD	0.97 ± 0.10	0.99 ± 0.13	0.034
IFOF	MD	0.95 ± 0.06	0.95 ± 0.15	0.254
Uncinate Fasciculus	MD	0.91 ± 0.06	0.94 ± 0.07	0.063
CST	MD	0.68 ± 0.19	0.67 ± 0.24	0.057
Cingulum anterior	MK	0.84 ± 0.06	0.83 ± 0.10	0.914
Cingulum posterior	MK	1.09 ± 0.07	1.09 ± 0.08	0.607
Cingulum hippocampus	MK	1.00 ± 0.08	1.01 ± 0.09	0.275
Fornix	MK	0.77 ± 0.08	0.79 ± 0.07	0.179
Corpus callosum	MK	0.90 ± 0.06	0.91 ± 0.06	0.397
SLF	MK	1.18 ± 0.05	1.17 ± 0.06	0.234
ILF	MK	1.02 ± 0.06	1.00 ± 0.12	0.454
IFOF	MK	1.01 ± 0.04	1.00 ± 0.16	0.970
Uncinate Fasciculus	MK	1.03 ± 0.05	1.02 ± 0.06	0.379
CST	MK	1.16 ± 0.06	1.14 ± 0.14	0.429

MD [10^-9 m^2/s]. Values are given as mean ± SD. ILF, inferior longitudinal fasciculus; IFOF, inferior fronto-occipital fasciculus; SLF, superior longitudinal fasciculus; CST, corticospinal tract; FA, fractional anisotropy; MD, mean diffusivity; MK, mean kurtosis; NDI *f*_iso_ and *f*_ic_, neurite density imaging measures.

^a^ *p*, Mann-Whitney U test.

^b^ *p* < .05, ANCOVA (age and gender adjusted).

P-values given in bold were significant when both using Mann-Whitney U test and ANCOVA.

**Table 4:** Neurite density imaging (NDI) measures in white matter structures in patients with Parkinson’s disease (PD) and healthy controls (HC).

Region	Parameter	HC (n = 44)	PD (n = 105)	*p*-value ^a^ (PD vs. HC)
Cingulum anterior	NDI *f*_ic_	0.58 ± 0.10	0.57 ± 0.07	0.340
Cingulum posterior	NDI *f*_ic_	0.71 ± 0.12	0.72 ± 0.06	0.582
Cingulum hippocampus	NDI *f*_ic_	0.72 ± 0.13	0.75 ± 0.07	0.658
Fornix	NDI *f*_ic_	0.71 ± 0.09	0.72 ± 0.1	0.472
Corpus callosum	NDI *f*_ic_	0.76 ± 0.13	0.76 ± 0.09	0.675
SLF	NDI *f*_ic_	0.72 ± 0.04	0.70 ± 0.09	0.351
ILF	NDI *f*_ic_	0.64 ± 0.05	0.62 ± 0.08	0.167
IFOF	NDI *f*_ic_	0.65 ± 0.04	0.64 ± 0.08	0.184
Uncinate Fasciculus	NDI *f*_ic_	0.64 ± 0.04	0.63 ± 0.06	0.225
CST	NDI *f*_ic_	0.73 ± 0.04	0.71 ± 0.06	**0.043** ^b^
Cingulum anterior	NDI *f*_iso_	0.11 ± 0.03	0.10 ± 0.03	0.268
Cingulum posterior	NDI *f*_iso_	0.10 ± 0.03	0.10 ± 0.03	0.484
Cingulum hippocampus	NDI *f*_iso_	0.14 ± 0.04	0.14 ± 0.04	0.923
Fornix	NDI *f*_iso_	0.50 ± 0.09	0.49 ± 0.11	0.724
Corpus callosum	NDI *f*_iso_	0.29 ± 0.04	0.29 ± 0.05	0.690
SLF	NDI *f*_iso_	0.11 ± 0.03	0.12 ± 0.03	**0.030** ^b^
ILF	NDI *f*_iso_	0.15 ± 0.04	0.16 ± 0.04	0.134
IFOF	NDI *f*_iso_	0.14 ± 0.03	0.15 ± 0.03	0.226
Uncinate Fasciculus	NDI *f*_iso_	0.13 ± 0.03	0.14 ± 0.03	0.112
CST	NDI *f*_iso_	0.11 ± 0.03	0.12 ± 0.03	0.060

Values are given as mean ± SD. ILF, inferior longitudinal fasciculus; IFOF, inferior fronto-occipital fasciculus; SLF, superior longitudinal fasciculus; CST, corticospinal tract; FA, fractional anisotropy; MD, mean diffusivity; MK, mean kurtosis; NDI *f*_iso_ and *f*_ic_, neurite density imaging measures.

^a^ *p*, Mann-Whitney U test.

^b^ *p* < .05, ANCOVA (age and gender adjusted). P-values given in bold were significant when both using Mann-Whitney U test and ANCOVA.

### Differences in dMRI parameters in PIGD compared to TD

The comparisons in GM structures between PIGD and TD are shown in Table 2. Compared to TD, patients with the PIGD phenotype exhibited a 7% increase in MD in the putamen (Mann-Whitney *U* test, *p* < .01). The difference remained significant after adjusting for age (ANCOVA, *p* < .05). There were no differences in dMRI parameters in the analyzed WM tracts between the two groups (data not shown).

### Correlations between clinical scores and dMRI parameters in PD

Next we analyzed correlations between the dMRI parameters that were changed in PD compared to HC (according to the analyses above) with clinical measures of motor and cognitive function.

**Fig 2:**
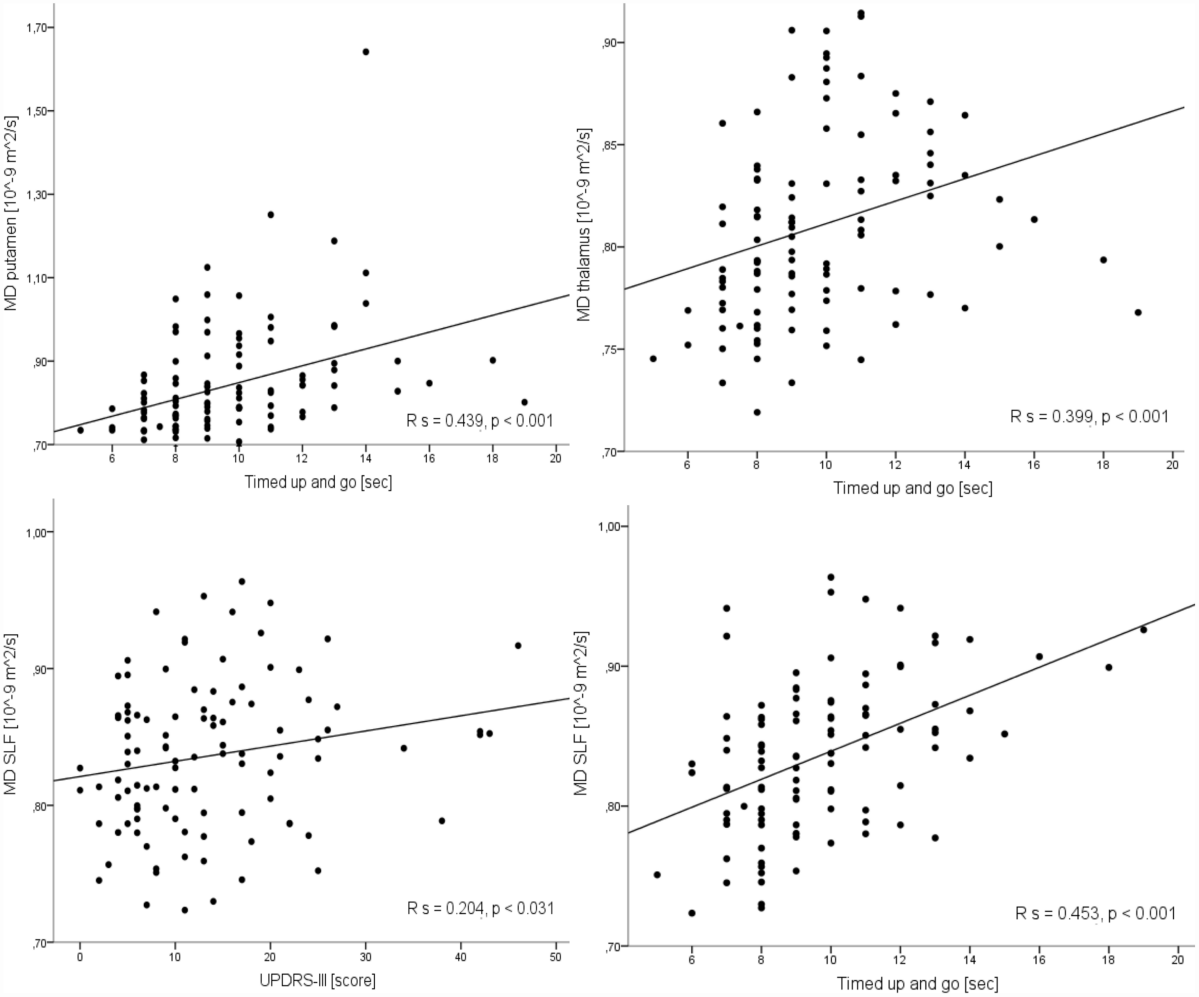
Correlation between mean diffusivity (MD) in the putamen, thalamus and superior longitudinal fasciculus (SLF) with the time up and go test score and between the MD in the SLF with Unified Parkinson’s disease rating scale motor part (UPDRS-III) score. A moderate positive correlation was found between MD in the putamen, thalamus and SLF and time up and go test score. A moderate positive correlation was found between the MD in the SLF and UPDRS-III scale score. R_s_, Spearman’s rho.

**Putamen:** Increased MD correlated significantly with a worse motor function, as measured by H&Y (R_s_ = .297, *p* < .005), UPDRS-III motor score (R_s_ = .192, *p* < .05), tandem gait (R_s_ = .439, *p* < .001), and timed up and go (R_s_ = .443, *p* < .001) tests (Fig 2).

MK correlated negatively with the disease duration (R_s_ = −.206, *p* < .05). Furthermore, increased MD correlated with impaired cognitive speed and attention, as measured by AQT (R_s_ = .219, *p* < .05). Decreased MK was associated with reduced memory performance, as measured by ADAS-Cog item 3 (R_s_ = .258, *p* < .01). In summary, a high MD and a low MK in the putamen are associated with more severe motor and cognitive symptomatology.

**Thalamus:** Increased MD correlated significantly with a worse motor speed and balance, as measured by the Hand test (R_s_ = −.243, *p* < .05), tandem gait and timed up and go tests (R_s_ = .222, *p* = .05 and R_s_ = .399, *p* < .001, respectively, Fig 2). Decreased FA also correlated negatively with timed up and go test (R_s_ = −.226, *p* < .05).

**SLF:** Increased MD correlated significantly with the worsening of the motor function, as measured by H&Y, UPDRS-III motor score, tandem gate, timed up and go tests and the cognitive function, as measured by AQT and ADAS-Cog (R_s_ = .204–358, *p* < .05). Increased *f*_iso_ correlated significantly with the worsening of the motor function, as measured by tandem gate and timed up and go tests and the cognitive function, as measured by ADAS-Cog (R_s_ = .205–325, *p <* .05). Increased *f*_iso_ correlated significantly with the worsening of the cognitive function as measured by MMSE (R_s_ = −.202, *p <* .05). In the caudate head and thalamus, *f*_iso_ did not correlate neither with disease duration nor with clinical scales.

### Differences in dMRI parameters in PD compared to PSP or MSA

To study whether the observed differences in dMRI parameters were specific for PD, we also investigated the same parameters in patients with PSP and MSA and compared these values to the ones obtained in the PD group. The results are given in table 5. In summary, only FA and MK of putamen seem to be specifically changed in PD where both these parameters are reduced indicating microstructural damage. All of the other changes observed in PD were also found to be changed in PSP and MSA in the same direction and often even more pronounced such as MD in the thalamus or putamen.

**Table 5:** Differences between patients with Parkinson's disease (PD) and cases with progressive supranuclear palsy (PSP) or multiple system atrophy (MSA).

	PD (n = 105)	PSP (n = 10)	MSA (n = 11)	*p*-value (PD vs PSP)	*p*-value (PD vs MSA)
Gender female: male	44: 61	5: 5	5: 6	1.000 ^a^	0.527 ^a^
Age (years)	66 ± 11	73 ± 6	63 ± 10	0.037	0.302
Disease duration (years)	5 ± 4	7 ± 2	6 ± 4	0.051	0.299
Hoehn and Yahr	2 ± 1	4 ± 1	4 ± 1	<0.001	<0.001
Schwab and England	90 ± 9	58 ± 35	67 ± 22	<0.002	<0.001
UPDRS-III motor score	13 ± 10	39 ± 14	34 ± 19	<0.001	<0.001
FA putamen	0.20 ± 0.03	0.23 ± 0.06	0.26 ± 0.03	0.053	**<0.001** ^c^
MD putamen	0.80 ± 0.14	0.88 ± 0.33	0.83 ± 0.15	0.275	0.276
MK putamen	1.14 ± 0.15	1.35 ± 0.25	1.34 ± 0.17	**0.012** ^b^	**0.001** ^c^
FA thalamus	0.36 ± 0.02	0.34 ± 0.04	0.35 ± 0.01	0.204	**0.034** ^c^
MD thalamus	0.80 ± 0.05	0.87 ± 0.11	0.84 ± 0.06	**0.044** ^b^	0.08
MD SLF	0.84 ± 0.05	0.89 ± 0.09	0.84 ± 0.04	**0.024** ^b^	0.984
NDI *f*_ic_ CST	0.71 ± 0.06	0.69 ± 0.07	0.71 ± 0.04	0.473	0.875
NDI *f*_iso_ SLF	0.12 ± 0.03	0.12 ± 0.01	0.13 ± 0.04	0.164	0.638

MD [10^-9 m^2/s]. Values are given as mean ± SD. UPDRS-III, Unified Parkinson’s disease rating scale motor part; FA, fractional anisotropy; MD, mean diffusivity; DKI, Diffusion Tensor Imaging; dMRI, diffusion magnetic resonance imaging; SLF, superior longitudinal fasciculus; CST, corticospinal tract; NDI *f*_iso_ and *f*_ic_, neurite density imaging measures; SD, standard deviation.

^a^ Pearson’s chi-square. *p*-value, Mann-Whitney U test.

^b^ PD/PSP,

^c^ PD/MSA,

*p* < .05, ANCOVA (age adjusted). P-values given in bold were significant when both using Mann-Whitney U test and ANCOVA.

### Diagnostic accuracy of the studied dMRI parameters in identification of PD

Based on the ANCOVA test, the univariate binary logistic regression analysis were performed in order to test the potential of dMRI parameters for the differential diagnosis of PD vs. HC. MD and MK in the putamen were included in the models, see Statistical analysis. A summary of the results is shown in S1 Table. Logistic regression analysis confirmed that the MD and MK in the putamen could significantly (*p* < .05) discriminate PD from HC. The sensitivity and specificity for these parameters, calculated using a ROC curve analysis, showed the optimal cutoff level of 0.79 for MD and of 1.18 for MK in putamen (with an area under the ROC curve of 0.62−0.65) to discriminate PD from HC with a sensitivity and specificity of around 60%.

## Discussion

We investigated disease-specific structural changes in deep grey matter and white matter in patients with PD. In this cohort with 105 patients with PD and 44 healthy controls, we found that MD in the thalamus was increased and FA was decreased in PD. These parameters correlated significantly with the worsening of motor speed and balance. In the putamen, MD was increased and FA and MK were decreased in PD. These changes were associated with worsening of motor speed, balance, and cognitive function. Further, in the white matter of PD patients, MD and *f*_iso_ in the SLF were increased and correlated significantly with worsening of motor speed, balance, and cognitive function. Further, we found that MD in the putamen was increased in PIGD compared to the TD. However, most changes except those of MD and MK in the putamen were not specific to PD but also found in MSA or PSP.

The present results confirm those of recent DTI studies showing that the increased MD and decreased FA in the thalamus and putamen can be found in patients with PD [12–16, 63]. Nevertheless, DTI studies have shown inconsistent results regarding the microstructural alterations in these regions, as there are some studies that showed no changes in dMRI measures in the striatum between controls and PD patients [17, 18]. Nicoletti et al. [17] investigated 16 patients with PD and 15 HC. Paviour et al. [18] investigated only 12 PD and 7 HC. These limited statistical power due to the small group sizes may explain why no effects were found in these studies. Szczepankiewicz et al. [64] showed that group sizes above 15 are required to detect effects of 5% with a power of 90% in large tract structures. Smaller ROIs, and lower SNR at 1.5 T, as was used in these studies, would lead to ever stronger requirements on group size to attain sufficient power. Thus these inconsistencies in the results may be due to differences in sample size and sample characteristics, as well as methodological differences between ROI analysis and voxel-based analysis. However, the present results indicate that there are indeed significant, although not very prominent, dMRI changes in the putamen and thalamus in PD, which may be related to progressive degeneration of nigrostriatal dopaminergic neurons. Our study did not find FA difference in any WM region between groups, and only found elevated MD and *f*_iso_ in SLF and reduced *f*_*ic*_ in CST in patients with PD compared to HC (Table 4). The reason for this discrepancy is not clear. That fact that our study did not find FA difference in any region between groups and only found the MD alteration is in line with the previous report that used Tract-Based Spatial Statistics (TBSS) analysis [63]. Other TBSS study reported only the FA alteration in WM in PD patients [65]. We therefore now caution the interpretation of these findings. In fact, the SLF is composed of 4 bundles of axons, connecting multiple frontal and prefrontal regions with superior temporal and parietal areas [66]. As a consequence of its structural heterogeneity, it is related to a range of premotor, motor, visuospatial, and auditory functions [66]. This probably can explain the clinical correlations we found with dMRI alterations in SLF. Previous studies have indeed found correlation between SLF and deficits in premotor functions and visuospatial perception in PD [67–70].

We did not find any clear diagnostic utility of measuring of MD and FA in the caudate nucleus, putamen, globus pallidus or substantia nigra when identifying PD. This finding is partly in agreement with Wang et al. [24]. Further, Kamagata et al [65], showed reduced MK in the frontal, parietal, occipital, right temporal white matter, posterior corona radiata and SLF in a PD group compared with a control group when using tract-based spatial statistics. these changes were not observed in the present study. However, the PD patients in the present study had less motor symptoms and/or shorter disease duration, which could explain these discrepancies. Considered together, the results might suggest that alterations of MK in basal ganglia and white matter probably might not improve the diagnostic work-up of early PD. However, such alterations may be are related to the disease severity of PD since we found that MK of putamen in PD patients correlate with disease duration and congnitive performance.

The clinical heterogeneity of PD is well recognized [6–8]. Early postural instability and gait involvement in PIGD dominant PD has been associated with a worse prognosis for motor and cognitive function, whereas the TD form of PD might be more benign [6–8]. Our results showed that MD in the putamen was increased in PIGD when compared to the TD. One possible explanation for the differences between the PIGD and TD might be the distinctly different patterns of neurodegeneration. In a study by Jellinger et al. [7] patients with PIGD demonstrated more severe cell loss in the ventrolateral part of SNpc, which projects to the dorsal putamen. One can speculate that pathology at the substantia nigra level could affect the discharge to the putamen as well as the degree of the subtle diffusion changes of putamen. Potentially 7 Tesla MRI that could assay more subtle changes in grey matter organization, and assessment of the alignment of fibers in white matter by novel dMRI techniques [64] might add some of the evidence. Even though we found several changes in dMRI in putamen, thalamus and SLF in PD, these changes were quite modest compared to controls and most of these changes were also observed in MSA or PSP. Consequently, we believe that dMRI of white matter tracts or the grey matter of the basal ganglia are unlikely to be used in the clinic to reliably detect early PD. However, it is intriguing that almost all dMRI abnormalities in PD correlate significantly with motor and cognitive function. Especially increased MD in putamen, thalamus and SLF was associated with worse motor functions (UPDRS-III motor score, tandem gate, and timed up and go test) as well as with worse cognitive function (MMSE, AQT, and memory delayed recall part of ADAS-cog), suggesting that better microstructural integrity as measured by diffusion MRI correlates with better clinical function. Several potential disease-modifying therapies for PD, like immunotherapy against α-synuclein, are currently being developed [71]. It will be very important to establish methods that objectively and reliably can tract the disease progression over time to be able to evaluate the effects of such treatments. Maybe dMRI in the basal ganglia could be included in such analyses, besides PET imaging of the integrity dopaminergic neurons, since the intersubject variability in dMRI parameters is much larger than the intrasubject variability [64]. However, longitudinal studies in PD evaluating the change in dMRI over 2–4 year is needed before such conclusions can be drawn.

The data reported in present study suggested that NDI measures in the basal ganglia are not useful for detection of PD patients. However, we did not assess NDI in the substantia nigra, because the low SNR in the substantia nigra was suspected of causing artificial changes in NDI. Using NODDI, Kamagata et al. [72] found lower *V*_ic_ (*f*_ic_ in our study) in the contralateral substantia nigra part compacta (SNpc) and putamen in PD patients. *V*_ic_ might be an indirect measure of nigrostriatal microstructural changes such as cell loss, morphological changes in dendritic length, and loss of dendritic spines and could be associated with disease severity in PD [72], indicating that NODDI in the substantia nigra might be useful for early diagnosis of PD as well as assessment of its subsequent progression. However, achieving sufficient SNR for accurate quantification of the neurite density in this iron-rich structure is challenging.

There are limitations with the study. The data was obtained in 2.3×2.3×2.3 mm^3^ voxels. The rather large voxel size implies that partial volume effects may be present in spite of efforts to avoid inclusion of surrounding structures during ROI placement. Another limitation was that the significance threshold was not corrected for multiple comparisons. This omission was motivated by the explorative nature of this study where we wanted to minimize the type II error rate. Furthermore, correcting for multiple comparisons would only strengthen the main conclusion that the magnitude of dMRI changes in PD are too small to be used to reliably detect early PD in a clinical setting. In addition, the analyses of disease stage-related microstructural changes dMRI is cross-sectional and need to be replicated and confirmed in longitudinal studies. Lastly, the ROIs were drawn manually by one author, and therefore the reproducibility of the measurements was uncertain. Rater bias was prevented by blinding, and the intra-rater variability coefficient was larger than 0.9 for all ROIs.

## Conclusions

The present study shows significant changes in dMRI parameters in the putamen, thalamus and SLF in PD. Most of the changes were modest in magnitude, and dMRI is unlikely to be useful in order to reliably detect early PD. However, changes in dMRI parameters correlated with worse motor and cognitive function in PD. Consequently, future longitudinal studies are needed to determine whether dMRI can be used to reliably track the disease progression of PD, and thereby might be of used in clinical trials, evaluating novel therapies with potential disease-modifying effects.

## Supporting Information

S1 Fig The correlation between the signal-to-noise ratio of globus pallidus relative to white matter and the mean diffusivity. PAL, globus pallidus; MD, mean diffusivity; SNR, signal-to-noise ratio. Lower values of MD were clearly associated to lower SNR.
(TIF)

S1 File Increased iron in substantia nigra reduces the signal-to-noise ratio. (DOCX)

S1 Table Use of DKI parameters in differential diagnosis. Mean diffusivity (MD, 10^9 mm^2/s) and mean kurtosis (MK), differentiating patients with Parkinson disease (PD) from healthy controls (HC); PD from patients with progressive supranuclear palsy (PSP) and multiple system atrophy (MSA). AUC, area under curve; DKI, diffusion kurtosis imaging; ROC, receiver operating characteristic analysis. *Significant differences between PD vs HC and PD vs PSP and MSA, p < 0.05, using binary logistic regression, adjusted for age and sex (PD vs HC) and adjusted for age (PD vs PSP and MSA).
(DOCX)
