# Supplementary figures and images for: Alterations of Diffusion Kurtosis and Neurite Density Measures in Deep Grey Matter and White Matter in Parkinson’s Disease
Source: PLoS One. 2016 Jun 30;11(6):e0157755. doi: 10.1371/journal.pone.0157755 (PMC4928807; doi:10.1371/journal.pone.0157755)

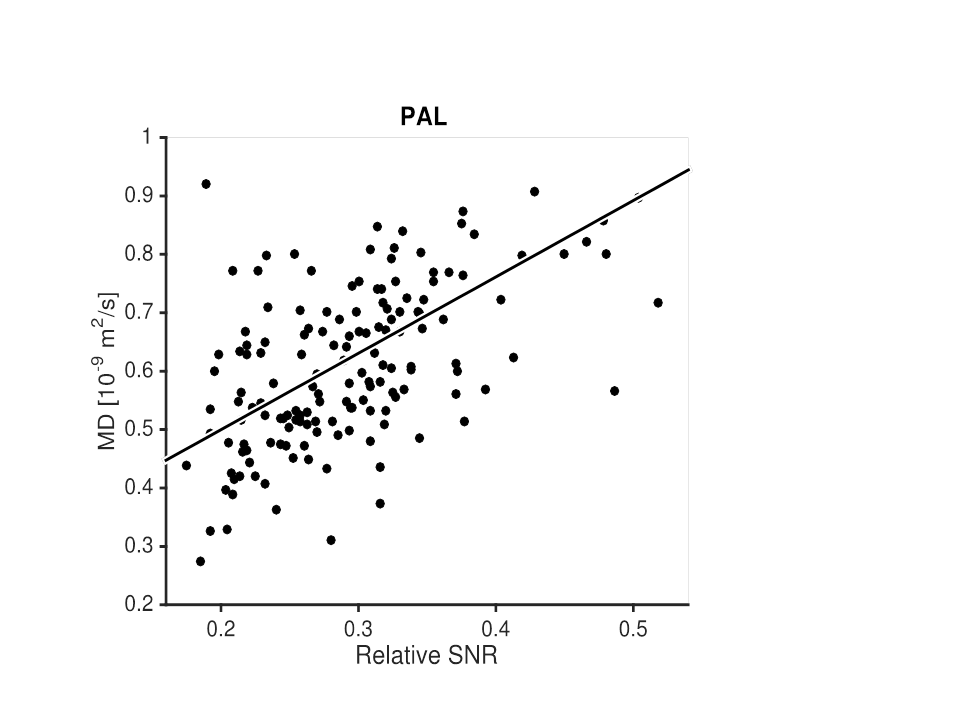

Supplement: S1 Fig — PAL, globus pallidus; MD, mean diffusivity; SNR, signal-to-noise ratio. Lower values of MD were clearly associated to lower SNR. (TIF) [file pone.0157755.s001.tif]
