# Supplementary material for: Alterations of Diffusion Kurtosis and Neurite Density Measures in Deep Grey Matter and White Matter in Parkinson’s Disease
Source: PLoS One. 2016 Jun 30;11(6):e0157755. doi: 10.1371/journal.pone.0157755 (PMC4928807; doi:10.1371/journal.pone.0157755)
Supplement: S1 File — (DOCX) [file pone.0157755.s002.docx]

# S2 Text. Increased iron in substantia nigra influence reduced signal-to-noise ratio.

Increased iron in substantia nigra can reduce the relaxation time and thus the lead to a substantially reduced signal-to-noise ratio (SNR). As a consequence, effects of noise can lead to elevated mean kurtosis (MK) and reduced mean diffusivity (MD) [Jones, 2004; Jensen, 2010]. To whether the SNR was sufficiently high for accurate Diffusion kurtosis imaging (DKI) of these structures, we investigated the SNR in grey matter (GM) of our DKI data. The SNR of various GM structures relative to that of white matter (WM) was calculated by normalizing the signal in each GM region by the average signal from all of the WM. In the thalamus, caudate nucleus, putamen, the SNR was 50–70 % of that in the WM. However, in the substantia nigra, red nucleus and globus pallidus the SNR was approximately 25–30% of that in WM. In the pons and midbrain, the SNR was approximately 50% of that in WM. Low SNR can result in higher MK and lower MD than expected. These effects can be explained by effects of the rectified noise floor [Jensen, 2010]. For the substantia nigra, red nucleus and globus pallidus, the correlation between MD and the normalized SNR was high (*r* > .5, p < 10^–9^ for all cases, S3 Fig). In the thalamus, caudate nucleus and putamen, no correlation was found. For this reason, the average SNR in each ROI and each group should be assessed before values are interpreted. We recommend to avoid analysis of DKI data in the substantia nigra, red nucleus and globus pallidus for data acquired with protocols optimized for WM imaging, as was the case for the present study. To optimize protocol for GM, the voxel size should be increased. However, larger voxel size will make difficult to localize substantia nigra and red nucleus with high precision, since these structures are small.
