# Supplementary material for: Alterations of Diffusion Kurtosis and Neurite Density Measures in Deep Grey Matter and White Matter in Parkinson’s Disease
Source: PLoS One. 2016 Jun 30;11(6):e0157755. doi: 10.1371/journal.pone.0157755 (PMC4928807; doi:10.1371/journal.pone.0157755)
Supplement: S1 Table — Mean diffusivity (MD, 10^9 mm^2/s) and mean kurtosis (MK), differentiating patients with Parkinson disease (PD) from healthy controls (HC); PD from patients with progressive supranuclear palsy (PSP) and multiple system atrophy (MSA). AUC, area under curve; DKI, diffusion kurtosis imaging; ROC, receiver operating characteristic analysis. *Significant differences between PD vs HC and PD vs PSP and MSA, p < 0.05, using binary logistic regression, adjusted for age and sex (PD vs HC) and adjusted for age (PD vs PSP and MSA). (DOCX) [file pone.0157755.s003.docx]

**S1 Table. Use of DKI parameters in differential diagnosis of patients with Parkinson’s disease from healthy controls.**

| **Structure** | **Parameter** | **AUC (ROC)** | **Cutoff** | **Sensitivity, %** | **Specificity, %** |
| --- | --- | --- | --- | --- | --- |
| Putamen* | MK | 0.62 | 1.18 | 58 | 59 |
| Putamen* | MD | 0.65 | 0.79 | 60 | 59 |

Mean diffusivity (MD, 10^-9 m^2/s) and mean kurtosis (MK). AUC, area under curve; DKI, diffusion kurtosis imaging; ROC, receiver operating characteristic analysis. *Significant differences between patients with Parkinson’s diseases from healthy controls, *p* < .05, using binary logistic regression, adjusted for age and sex.
